# Characterization of 3D printing techniques: Toward patient specific quality assurance spine-shaped phantom for stereotactic body radiation therapy

**DOI:** 10.1371/journal.pone.0176227

**Published:** 2017-05-04

**Authors:** Min-Joo Kim, Seu-Ran Lee, Min-Young Lee, Jason W. Sohn, Hyong Geon Yun, Joon Yong Choi, Sang Won Jeon, Tae Suk Suh

**Affiliations:** 1 Department of Biomedical Engineering, Research Institute of Biomedical Engineering, College of Medicine, The Catholic University of Korea, Seoul, Korea; 2 Department of Radiation Oncology, Yonsei Cancer Center, Yonsei University College of Medicine, Seoul, Korea; 3 Department of Radiation Oncology, School of Medicine, Case Western Reserve University, Cleveland, Ohio, United States of America; 4 Department of Radiation Oncology, College of Medicine, DongGuk University Hospital, Goyang, Korea; Seoul National University College of Medicine, REPUBLIC OF KOREA

## Abstract

Development and comparison of spine-shaped phantoms generated by two different 3D-printing technologies, digital light processing (DLP) and Polyjet has been purposed to utilize in patient-specific quality assurance (QA) of stereotactic body radiation treatment. The developed 3D-printed spine QA phantom consisted of an acrylic body phantom and a 3D-printed spine shaped object. DLP and Polyjet 3D printers using a high-density acrylic polymer were employed to produce spine-shaped phantoms based on CT images. Image fusion was performed to evaluate the reproducibility of our phantom, and the Hounsfield units (HUs) were measured based on each CT image. Two different intensity-modulated radiotherapy plans based on both CT phantom image sets from the two printed spine-shaped phantoms with acrylic body phantoms were designed to deliver 16 Gy dose to the planning target volume (PTV) and were compared for target coverage and normal organ-sparing. Image fusion demonstrated good reproducibility of the developed phantom. The HU values of the DLP- and Polyjet-printed spine vertebrae differed by 54.3 on average. The PTV D_max_ dose for the DLP-generated phantom was about 1.488 Gy higher than that for the Polyjet-generated phantom. The organs at risk received a lower dose for the 3D printed spine-shaped phantom image using the DLP technique than for the phantom image using the Polyjet technique. Despite using the same material for printing the spine-shaped phantom, these phantoms generated by different 3D printing techniques, DLP and Polyjet, showed different HU values and these differently appearing HU values according to the printing technique could be an extra consideration for developing the 3D printed spine-shaped phantom depending on the patient’s age and the density of the spinal bone. Therefore, the 3D printing technique and materials should be carefully chosen by taking into account the condition of the patient in order to accurately produce 3D printed patient-specific QA phantom.

## Introduction

Nowadays, advanced radiotherapy such as stereotactic body radiation therapy (SBRT) delivered high radiation dose into a small size of the tumor region using highly elaborated radiation fluence, and thus, patient-specific quality assurance (QA) plays an important role [1, 2]. Tumors of the spine are an especially common malignancy, and spinal tumors could cause neurological disabilities including pain. The SBRT could be performed in patients with spinal tumors, and the target volume for radiation treatment such as spine SBRT unavoidably includes the spinal cord during the radiation treatment planning process [3–5]. Thus, spine SBRT requires the inclusion of a steep dose gradient in delivered dose distribution, high prescription dose, small size of radiation fields, and extra image guidance [1, 6] to exclude the spinal cord from the delivered dose distribution. To verify and increase the accuracy of spine SBRT and also most of the radiation treatment, the QA process using a specialized patient-specific phantom has become increasingly important since patient-specific QA using a highly customized patient-specific phantom could clearly determine the accuracy of radiation treatment planning [7, 8].

Accurate and cost-effective production of these patient-specific phantoms requires the use of the latest technology, the three-dimensional (3D) printing technique. The advent of 3D printers has led to a growing interest in various fields. In particular, the characteristics of 3D printing, such as the versatility and variety of materials for 3D printing as well as the ability to customize products with the desired geometrical features are being promoted to utilize this latest technology in various fields and these merits of 3D printing have been recently integrated into the field of medical physics, especially in the development of bolus, compensators, and QA phantoms.

Various types of 3D printing technologies are available to researchers, including stereolithography (SLA or SL), fused deposition modelling (FDM), selective laser sintering (SLS), Polyjet printing, and digital light processing (DLP) [9]. SLA was the first 3D printing technology to be developed and it involves focusing a concentrated beam of ultraviolet (UV) light on the surface of a vat filled with a liquid photopolymer [10]. Recently, Bache et al. made a positive mold in the shape of a rodent using this technology [11]. FDM is also a relatively old 3D printing technology; however, and it is commonly used due to its lowest cost. This type of 3D printing uses thermoplastic materials such as acrylonitrile butadiene styrene (ABS, density 1.05 g/cm^3^) or poly lactic acid (PLA, density 1.25 g/cm^3^) [12]. There are a few papers involving FDM 3D printing and these studies have resulted in the development of various types of phantoms for application in the field of medical physics such as the development of a phantom including soft-tissue [7], low-density materials to simulate the patient’s lung [13–15], and in magnetic resonance imaging (MRI) for breast phantoms [16]. The advanced printing method is SLS, which involves the use of a high-power laser to fuse plastic or metal powders into the desired 3D shape [17]. Madamesila et al. evaluated low-density phantoms by comparing FDM and SLS [14]. Lastly, DLP and Polyjet printing techniques appear to be appropriate for construction of patient-specific QA phantoms consisting of high-density materials with high output resolution. There have been a few publications regarding the Polyjet technique: Design of a patient-specific phantom for liver [18], neurovascular model [19], imaging phantom for the evaluation of a new CT reconstruction algorithm [20, 21]. On the other hand, to the best of our knowledge, the DLP printing technique has never been used in the medical physics field.

While 3D printing has been utilized in various fields to produce highly customized objects as described above, the phantom developed from the 3D printing technique is available only for the soft tissue or the applied low-density materials in the medical physics field. In this work, 3D printed objects that mimic the human spine using high-density materials with DLP and Polyjet printing techniques were produced and each characteristic of these two different patient-specific spine phantom sets was investigated by comparing treatment radiation doses and Hounsfield units (HUs) using the corresponding CT image sets.

## Materials and methods

### 3D printer technology

Two types of 3D printers were used in this study: a DLP printer and a Polyjet printer. The DLP printing technology uses a more conventional light source, such as an arc lamp with a liquid-crystal display panel or a deformable mirror device which is applied to the entire surface of the vat of photopolymer resin in a single pass. The DLP printing technique also produces highly accurate parts with excellent resolution. The main advantages of this technique are that DLP technique can harden a whole layer in a fraction of the time and it takes to a laser to trace around and fill in each item on the print bed. With use of this production method, DLP can provide higher printing speeds at a relatively low cost [22]. The Polyjet 3D printer is similar to an ink jet printer; however, this technique applies resins instead of ink. The resin is laid down on a print bed layer by layer and then it is hardened using UV light. Some Polyjet machines can make a combination of hybrid materials because of their multiple print heads. Printing materials used for Polyjet technique are very diverse, ranging from hard plastics to soft rubber [23].

The characteristics of each printer used in this study are summarized in Table 1. For the DLP technique, Titan 1 3D printer manufactured by Kudo3D (Pleasanton, CA, USA) was used in this investigation. This printer yields resolution values of 30 to 70 μm in the x and y directions, and a resolution of 5 μm in the z-direction [22]. In case of the Polyjet technique, the phantom was printed using an Objet Connex 3D printer (Objet Geometries, Rehovot, Israel), which provides a z resolution of 16–30 μm and accuracy of 20–80 μm [24]. For both technology, the same main material, acrylic polymer with a density of 1.29–1.39 g/cm^3^ [25], was applied and the density of this acrylic polymer is most similar to the density of the human spine. [26, 27]

**Table 1:** The characteristics of the Polyjet and digital light projection (DLP) 3D printers.

3D Printer	Sample Hardware	Accuracy	Resolution (z)	Reference
Polyjet	Object Connex	20–85 µm	16–30 µm	[22]
DLP^a^	Kudo 3D, Titan1	6.35 µm	5 µm	[24]

DLP^a^: digital light processing

### Spine modelling process for 3D printing

**Fig 1:**
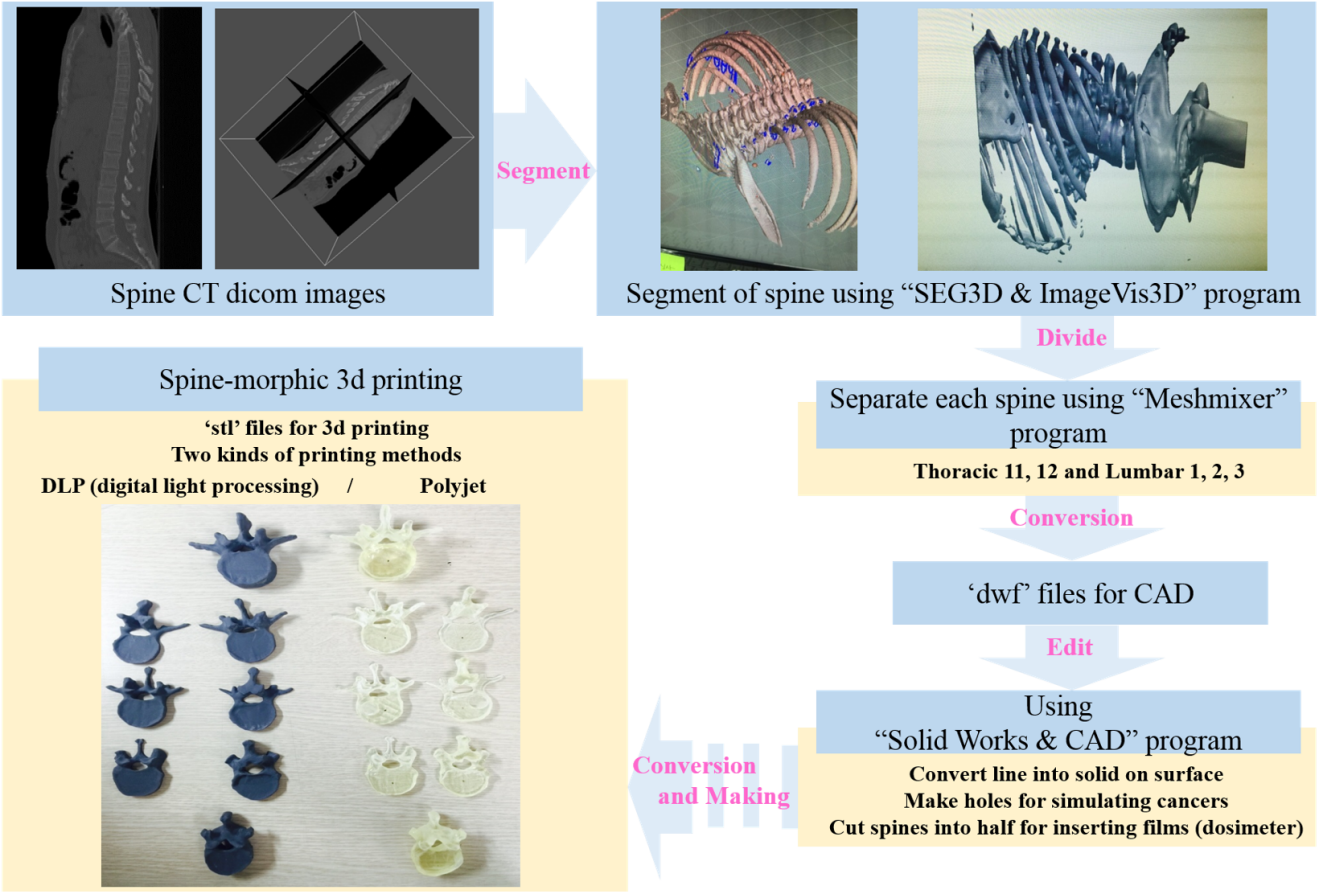
Diagram of the workflow framework that we followed for producing 3D printed spine-shaped phantom from CT data.

Fig 1 shows the workflow for 3D printing to generate the human spine. Volumetric CT data were exported as 1.25 mm axial slices in the DICOM (.dcm) format to Seg3D and ImageVis3D (Scientific Computing and Imaging Institute, University of Utah, Salt Lake City, UT, USA), which are open-source software packages for volumetric image segmentation, volume rendering, and visualization. The spines were segmented and a 3D iso-surface was generated for each spine by generating a series of triangles using the above two software packages which can convert and export the stereolithography (STL) file format. The STL files were imported into Autodesk Meshmixer software (Autodesk Inc., San Rafael, CA, USA) to divide each spine and to convert the files into design web format (DWF) file formats. The DWF files were imported into SolidWorks 3D CAD software (SolidWorks Corp., Concord, MA, USA) to simulate spinal cancer by producing holes on the first lumbar vertebra (L1). The edited files were exported in the STL file format. Lastly, the twelfth thoracic vertebra (T12), L1 with tumors, and second lumber vertebra were divided laterally exactly into half using the Autodesk Meshmixer software to possibly measure the doses delivered to the inside of the vertebrae by inserting a dosimetric film.

### Development of a patient-specific spine QA phantom

**Fig 2:**
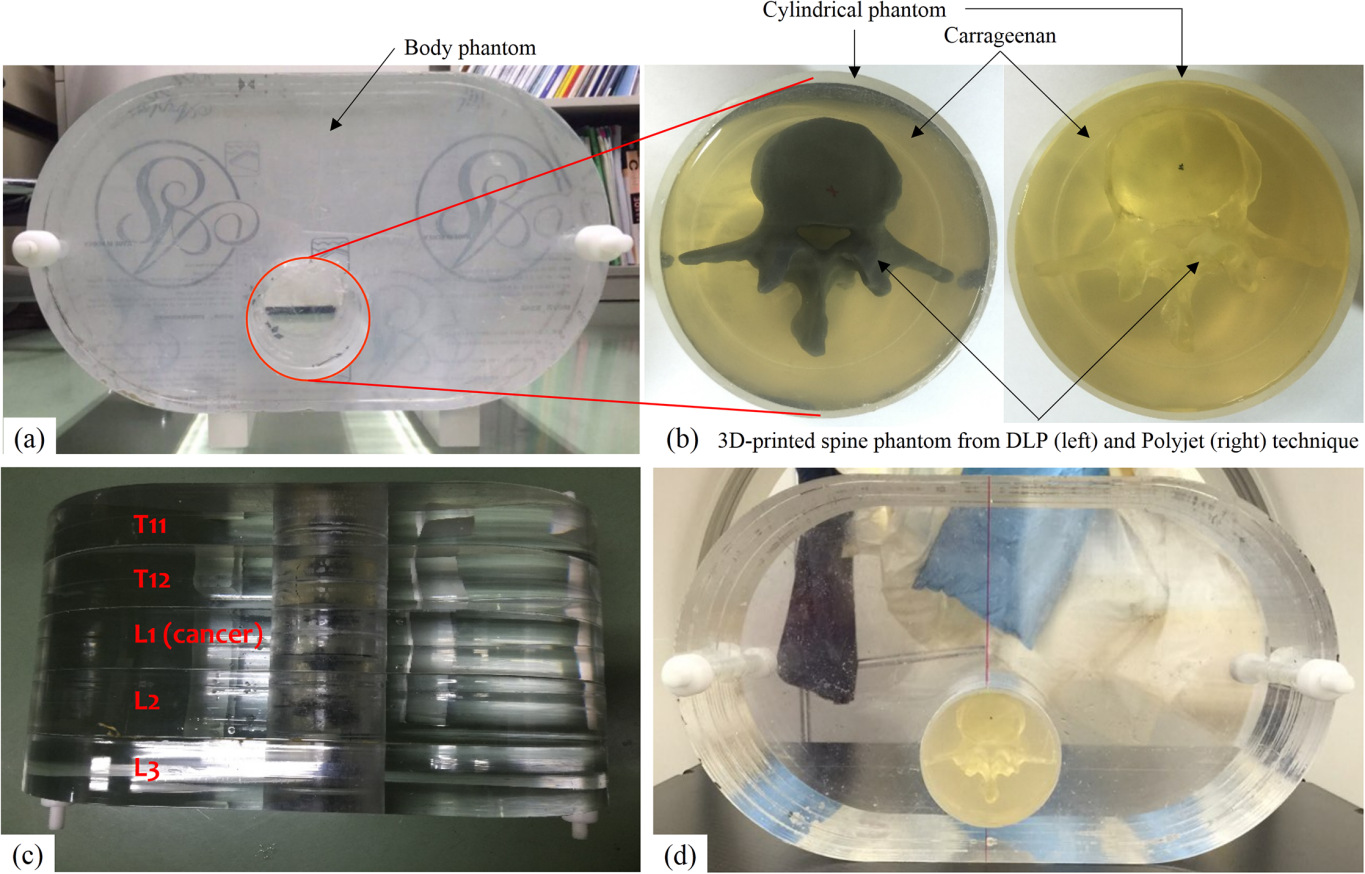
(a) Acrylic body phantom (b) 3D printed spine phantom generated by the DLP technique (left) and the Polyjet technique (right) fixed to a cylindrical phantom with carrageenan (c) Top view of the developed 3D printed spine quality assurance (QA) phantom, which consists of five slabs (d) Front view of the developed 3D printed spine QA phantom

The body phantom was constructed with an acrylic and it was the size of the human abdomen on average [28], and it consisted of five slabs and the gap between two slabs would be utilized for subsequent application by inserting dosimetry films. Since the human spine is located 2 cm from the back on average, the body phantom was also punctured at intervals of 2 cm from the back to insert a cylindrical phantom which contains the 3D printed spine. Carrageenan, a substance applied to MRI phantoms [29, 30], was used to fix the 3D printed spine phantom in the cylindrical phantom. In the pre-study results, a 1% concentration of carrageenan yielded an average HU of 8–10 on CT images and it was demonstrated that this concentration of carrageenan could simulate the spinal cord and cancer in patient-specific QA phantoms. Also, carrageenan at this concentration could become solidified after a certain period of time within 1 hours. Each spinal canal of the 3D printed spine was aligned at the centre of the cylindrical phantom. The two different sets of the 3D printed spine phantom created by the DLP and Polyjet printing technique were able to replace each other in the body phantom and these two different 3D printed spine phantoms inserted in the body phantom were called the DLP printed phantom and the Polyjet printed phantom in our study. Fig 2 shows the corresponding developed phantom images.

To evaluate the reproducibility and applicability of the developed SBRT QA phantoms and to compare the HU values of two different 3D printed spine phantoms, image fusion and calculation of HU values between the two different CT image sets were performed. MIM software (MIM Software, Cleveland, OH, USA) for image fusion was used. To measure the HU values, ovals of the same size (200 mm^2^) were drawn at the same location of the body of the 3D-printed spine phantom from T11 to L3 on each CT image, and then, the averages and standard deviations were calculated using the open-source program ImageJ (http://rsb.info.nih.gov/ij/).

### Treatment planning and evaluation

To investigate the acceptability of the developed SBRT QA phantom for purpose of radiation dosimetry, radiation treatment planning, dose calculation was performed. The developed phantom was scanned using a CT simulator (BrightSpeed, GE Healthcare, Fairfield, CT, USA) with a slice thickness of 1.25 mm and at 120 kVp, 200 mAs, 120 mA, a 50 cm field of view, and a 512ⅹ512 image matrix, which resulted in a voxel size of 0.977ⅹ0.977ⅹ1.5 mm using an axial acquisition. In our study, all treatment plans were generated using the Eclipse treatment planning system (Varian Associates, Palo Alto, CA, USA). All planning systems used a 2 mm dose grid for dose calculations and 6 MV photon beams, and dose calculation was performed by using the pencil beam convolution. The clinical target volume (CTV) was contoured, and a planning target volume (PTV) was created by adding a 3 mm margin. The spinal cord planning risk volume (PRV) was generated by adding a 2 mm margin to the delineated spinal cord [31, 32]. The PRV never overlapped with the PTV. The spinal cord and partial spinal cord PRV was limited to 5 mm above and below the PTV [33]. The PTV margin and spinal cord PRV were reflected in set-up errors and motion errors on spine SBRT [33].

A single-fraction SBRT treatment plan was developed using seven static 6-MV beams in a fixed gantry and sliding-window intensity-modulated radiation treatment (IMRT). All plans included a prescribed dose of 16 Gy to the PTV with an aim that at least 90% of the PTV received more than the prescribed dose and that the following dose constraints were satisfied: a maximum PTV dose of 23 Gy, a maximum spinal cord dose of 10 Gy, and a maximum partial cord PRV dose of 14 Gy [34].

To evaluate the SBRT QA plan, dose volume histograms were constructed. For the PTV, D_max_, D_mean_, D_95%_, and the relative volume of the PTV receiving at least 16 Gy (V_16Gy_) were estimated. The conformity index (CI) evaluates the appropriateness of the PTV for the prescription isodose volume in the treatment plans. The calculation for the CI is shown below [33].

$$CI=\frac{V_{PTV}\times V_{TV}}{TV^{2}{}_{PV}}$$

For the spinal cord, PRV, D_max_, and the relative volume of the spinal cord receiving at least 10 Gy (V_10Gy_) were evaluated.

## Results

### Development and evaluation of the patient-specific spine QA phantom

**Fig 3:**
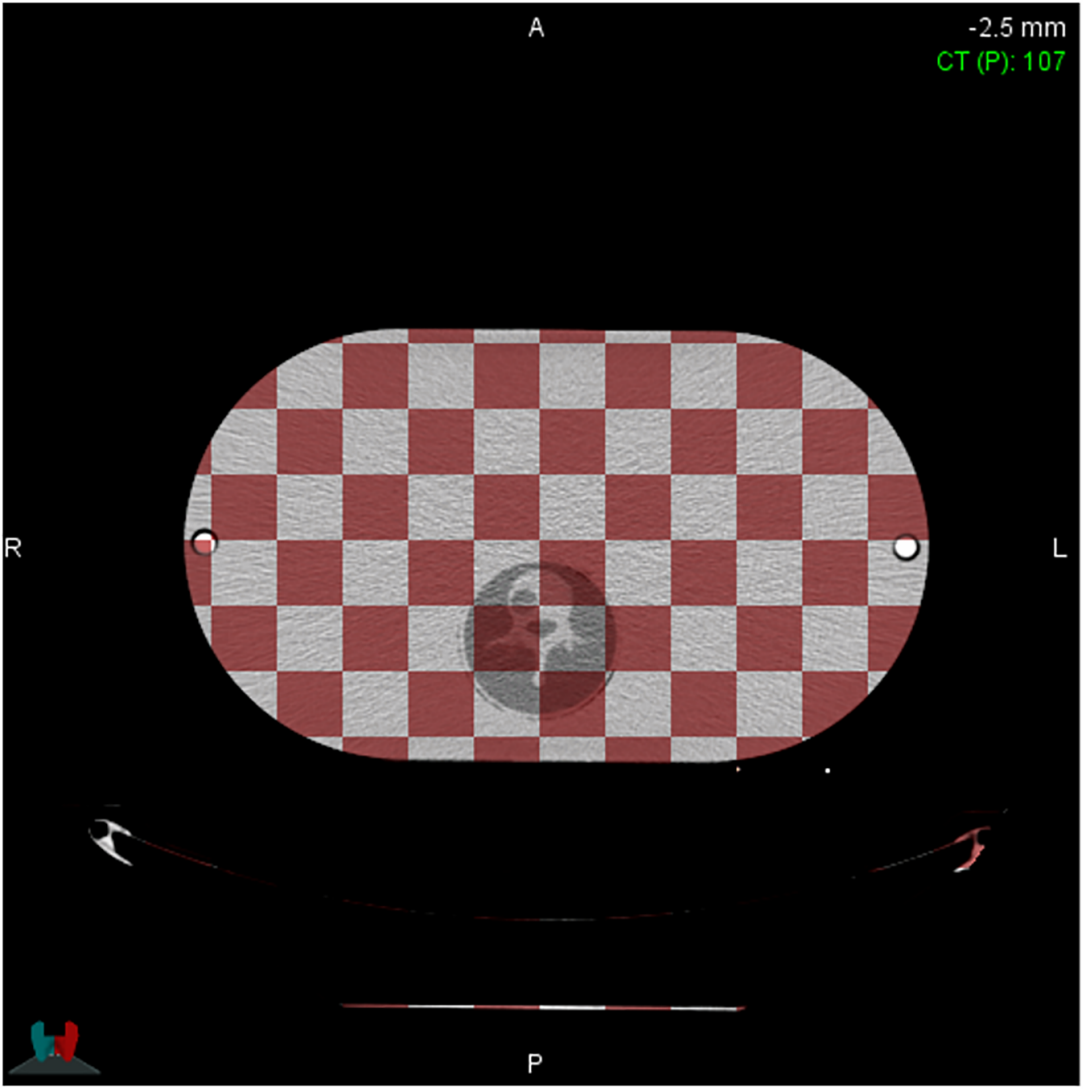
The result of image fusion.

Since the developed phantom is a body phantom with a form of replacing 3D printed spine phantoms which were made by using the DLP and Polyjet 3D printing methods, the ability to reproducibly manufacture phantoms is important. Reproducibility of the developed phantoms was verified by the evaluating the degree of overlap for the two different 3D printed spine phantom sets. The fused CT image sets of 3D printed L1 spine phantom which has the hole at vertebra body for simulation of spine tumor showed in Fig 3. The centers and the edge of 3D printed spin phantom were well matched each other without any distortion on the fused CT image set. Especially, vertebral foramen and the holes at vertebra body of 3D printed spine phantom on the fused CT image sets were also well matched as shown in Fig 3. Also, the CT images of carrageenan surrounding the spine were homogeneous in all slices and showed HU values of 8–10, as already observed in our pre-study. The mean HU values and standard deviations of carrageenan surrounding the Polyjet-printed spine and the DLP-printed spine were 8.98±1.05 and 10.88±1.67, respectively.

Table 2 summarizes the means and standard deviations of the HU values at the vertebral body from our results and it also presents these values according to the age of the patient and the type of printing technique from the study by Lee [26]. The difference between the HU values of the two 3D printed spine vertebrae (DLP and Polyjet) was 54.3 on average.

**Table 2:** The mean and standard deviation of the HU values from the spine body for the different 3D printing techniques and for the different patient ages.

Types of spine	Hounsfield units (means±standard deviation)	Reference
DLP^a^	152.1±3.5	
Polyjet	97.8±3.3	
40 to 49 years	175.0±48.0	[26]
50 to 59 years	150.2±40.4	
60 to 69 years	97.5±39.7	
70 to 79 years	81.0±32.0	
Over 80	51.8±32.7	

DLP^a^: digital light processing

### Dosimetric results of the patient-specific spine QA phantom according to the 3D printing technology

The radiation treatment planning results of the two phantom sets applied to the same SBRT plan is shown in Table 3. All plan results of both phantoms satisfied the dose criteria described in the ongoing RTOG 0631 study [34]. For the spinal cord PRV, the RTOG 0631 criteria stated that D_max_ should be less than 14 Gy, and the D_max_ values for both the Polyjet and DLP printed phantom sets were indeed smaller than this value. However, the D_max_ for treatment planning using the DLP printed phantom image was about 1.488 Gy higher than that for treatment planning using the Polyjet method. The conformity index values for dose distribution using the DLP and Polyjet phantom image sets were 0.957 was 0.962, respectively.

**Table 3:** Summary of the dosimetric results for the planning target volume (PTV) and organs at risk (OAR) for treatment planning using two different 3D printed phantom image sets.

	Dose Criteria	Planning using DLP^a^ printed phantom	Planning using Polyjet printed phantom
PTV			
D_max_(Gy)	<23	18.400	16.912
D_mean_(Gy)	-	17.008	16.336
D_95%_(Gy)	-	16.034	15.572
V_16_(%)	≥90.0	95.672	90.182
Conformity Index		0.957	0.926
PRV spinal cord			
D_max_(Gy)	≤14.0	13.758	13.923
V_10_(%)		13.977	23.332
Spinal cord			
D_max_(Gy)	≤10	9.700	9.900

DLP^a^: digital light processing

**Fig 4:**
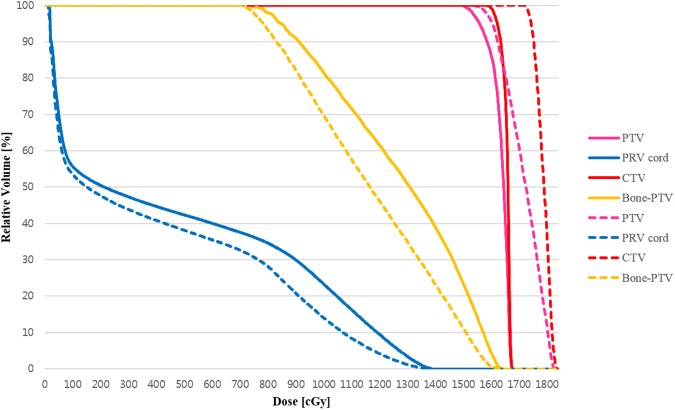
Dose volume histogram for the planning target volume (PTV) and organs at risk (OAR) from 3D printed spine phantom sets (solid line for digital light projection (DLP) printed phantom, and dotted line for Polyjet printed phantom) from spine stereotactic body radiation therapy (SBRT) treatment plans.

Fig 4 shows the graph of dose to relative volume for the CTV, PTV, spinal cord PRV, and spine without PTV. Even the same plan technique was applied for the DLP and Polyjet printed phantom image sets, the PTV and CTV doses were greater for the DLP printed phantom plan than for the Polyjet printed phantom plan. The DLP printed phantom delivered lower doses to the organs at risk (OAR) than the Polyjet printed phantom, as shown in Fig 4.

## Discussion

Even though anatomical phantoms are becoming more advanced, they have several limitations such as high cost and they are not fully customized for each patient [18]. However, with initiation of several 3D printing agencies, customers will soon be able to order conveniently and inexpensively produced patient-specific phantoms on demand. Therefore, the application of 3D printing technology in the medical field including the radiation treatment field has received focused attention. In this study, the applicability of patient-specific QA phantoms which simulated the human spine, one of the anatomic structures, the representative organ having a relatively high density than the other organs, was evaluated by generating 3D printed spine phantoms using two 3D printing techniques, DLP and Polyjet.

The developed phantom consists of a main body phantom and a cylindrical phantom to be inserted into the body phantom, and a 3D printed spine phantom was placed at the centre of the cylindrical phantom. This type of developed phantom that depends on the insertion method could cause a misalignment issue during the fabrication of the phantom. Thus, evaluation of the reproducibility in the fabrication of a patient-specific 3D printed QA phantom is important before using the developed phantom. As shown in Fig 3, there was neither image distortion nor inhomogeneity, and this result could be interpreted as follows: 3D printed spines were well fixed within carrageenan and the concentration of carrageenan was appropriate to cause solidification. These results demonstrated that 3D printed spine phantoms within body phantoms could be applied to different patients with good reproducibility.

The DLP and Polyjet technologies using an acrylic polymer are applicable to simulation of high-density organs because both these technologies use high density materials and produce stiff objects without formation of air bubbles inside the phantom. However, there are significant differences between the HU values for the two printed phantom image sets and the difference between the HU values of the 3D printed spine QA phantoms from the two printing methods in the current investigation was 54.3, which could actually cause a difference in the delivered radiation dose. This difference in the HU value depending on the 3D printing technique in spite of the application of the same printing material has been discussed below. First, additional materials were used for the Polyjet printing method. The Polyjet technology utilized a material consisting of an acrylic polymer and additional materials, including TangoPlus (Stratasys, MN, USA) and VeroWhite (Stratasys, MN, USA), to create a translucent object. On the other hand, the material used for the DLP technology contained only an acrylic polymer [20]. However, this effect may be negligible since only small quantities of these additional materials were used for Polyjet printing. Second, different printing techniques may result in products with different degrees of solidity or stiffness. Thus, different HU values could be obtained. The solidity of the product is related to the resolution of the printing technique in the z-direction. The DLP technique affords a higher resolution than the Polyjet technique, as shown in Table 1. In other words, the HU values resulting from the DLP printed phantom are also higher than the HU values resulting from the Polyjet printed phantom, which implies that products obtained with use of the former technique are relatively stiff. However, dependence of this different HU value on the 3D printing technique could be a helpful approach to produce the most highly customized patient-specific spine QA phantom. As shown in Table 2, the HU value of a vertebral body of a patient between 50 and 59 years of age was similar to the HU value of a DLP printed phantom, and the HU values for a patient between 60 and 69 years of age were well matched with the HU values of a Polyjet printed phantom. [26] Furthermore, the related research had reported that the average HU value of spine depending on different age groups decreased as the patients’ age increased and the differences in HU values from L1 to 4th Lumbar were significant among different age groups. [27, 35, 36] Therefore, our finding suggests that each 3D printing technique has its own special advantage in terms of the HU value that depends on the patient’s age and it also demonstrated that careful selection of the 3D printing technique and printing materials is required since even when the same material, which is known to have a similar density to the human spine, was applied for 3D printing, the calculated HU values from two different 3D printed spine phantom sets were different.

The dosimetric results from the two types of phantom image sets using the SBRT plan are shown in Fig 4 and Table 3. The dosimetric results demonstrated that the both 3D printed spine phantom could be applied in patient specific QA process since both dosimetric results were within a dose criteria guided by RTOG 0631 for all calculated dose index (Dose to PTV, PRV spinal cord, spinal cord) without unexpected dose gradient within phantom body and peripheral region of spine phantom and shown acceptable conformity index even the HU value of 3D printed spine phantom was slightly different. Furthermore, as shown in Fig 3, the good reproducibility of 3D printed spine phantom could be able to produce appropriate spine phantoms for therapeutic radiation purposes.

A limitation of our study was that the CT image set for the 3D printed spine phantom modelling was not acquired from a real patient with a spinal tumor and the CT data set for 3D printing modelling process was acquired from a normal patient. However, the main purpose of our study was to investigate the 3D printing technology for development of a patient-specific QA phantom for cases involving the spine, which is known as the organ having a relatively high density, and to compare the 3D printing techniques. For this purpose, the CT image from a normal patient, who has a standard shape of the spine and homogeneous composition, was useful for the image modelling process and 3D printing.

## Conclusions

In conclusion, this study confirmed that a 3D-printed phantom simulating a high-density (about 1.4 g/cm^3^) organ can be created based on CT images and that a developed 3D printed spine phantom could be utilized in patient-specific QA for SBRT. Additionally, a careful decision regarding the appropriate printing technique according to the patient’s condition is required since there was a difference in the HU value of about 54.3 following application of different printing technologies (DLP and Polyjet) even though the same material which has the same density had been utilized. In further studies, our methods will be applied to CT images of patients with actual spinal tumors and the appropriate printing technique and materials suggested in our results will be used for patient-specific QA for spine SBRT including dosimetric measurements to carry out an end-to-end test in order to increase the accuracy of spine SBRT.

## Supporting information

S1 File https://figshare.com/s/3ce045497691c13cf1fc. (DOCX)
