# Supplementary material for: Characterization of 3D printing techniques: Toward patient specific quality assurance spine-shaped phantom for stereotactic body radiation therapy
Source: PLoS One. 2017 May 4;12(5):e0176227. doi: 10.1371/journal.pone.0176227 (PMC5417437; doi:10.1371/journal.pone.0176227)
Supplement: S1 File — (DOCX) [file pone.0176227.s001.docx]

**S1 File:**

<https://figshare.com/s/3ce045497691c13cf1fc>
